# Disability associated with exposure to traumatic events: results from a cross-sectional community survey in South Sudan

**DOI:** 10.1186/1471-2458-13-469

**Published:** 2013-05-14

**Authors:** Touraj Ayazi, Lars Lien, Arne Henning Eide, Rachel Jenkins, Rita Amok Albino, Edvard Hauff

**Affiliations:** 1 Institute of Clinical Medicine, Faculty of Medicine, University of Oslo, Blindern, P.O box 1171, Oslo, 0318, Norway; 2 National Center for Dual Diagnosis, Innlandet Hospital Trust, Furnesvegen 26, Brumunddal, 2380, Norway; 3 SINTEF, Technology and Society, Pb. 124 Blindern, Oslo, 0314, Norway; 4 Department of Health Services and Population Research, King’s College London, Institute of Psychiatry, David Goldberg Centre, De Crespigny Park, London, UK; 5 Ahfad University for Women, P.O. Box 167, Omdurman, SUDAN; 6 Division of Mental Health and Addiction Department of Research and Development, Oslo University Hospital, Ulleval, Kirkeveien 166, Building 20, Oslo, 0407, Norway; 7 Faculty of Public Health, Hedmark University College, P.O.Box 400, Elverum, 2418, Norway

**Keywords:** Disability, Traumatic events, Post-conflict, South Sudan

## Abstract

**Background:**

There is a general lack of knowledge regarding disability and especially factors that are associated with disability in low-income countries. We aimed to study the overall and gender-specific prevalence of disability, and the association between exposure to traumatic events and disability in a post-conflict setting.

**Methods:**

We conducted a cross-sectional community based study of four Greater Bahr el Ghazal States, South Sudan (n = 1200). The Harvard Trauma Questionnaire (HTQ) was applied to investigate exposure to trauma events. Disability was measured using the Washington Group Short Measurement Set on Disability, which is an activity-based scale derived from the WHO’s International Classification of Disability, Functioning and Health.

**Results:**

The estimated prevalence of disability (with severe difficulty) was 3.6% and 13.4% for disability with moderate difficulties. No gender differences were found in disability prevalence. Almost all participants reported exposure to at least one war-related traumatic event. The result of a hierarchical regression analysis showed that, for both men and women, exposure to traumatic events, older age and living in a polygamous marriage increased the likelihood of having a disability.

**Conclusions:**

The finding of association between traumatic experience and disability underlines the precariousness of the human rights situation for individuals with disability in low-income countries. It also has possible implications for the construction of disability services and for the provision of health services to individuals exposed to traumatic events.

## Background

Individuals with disability are particularly vulnerable in conflict settings [1] and have amplified difficulties throughout the displacement process [2]. Women with disability are particularly vulnerable and face significant barriers in access to education, participation in social life, entering the open labour market, and access to health services [3]. Armed conflicts result in an increased number of disabled individuals, and people with disability have, in turn, an increased risk of becoming victims of violence because of their disability [4]. Nonetheless, data and relevant research on disability in developing countries are limited [5,6]. Estimated prevalence figures vary widely and comparability is highly problematic because standardised measures of disability have not been implemented [7]. Furthermore, the definition of disability has, over the years, changed from being impairment-based to being activity-based, with direct consequences on operationalisation and screening for disability [7]. One review of household surveys from nine developing countries indicated a disability prevalence of 1–2% [8], whereas another study [9] estimated the prevalence rate of individuals with disability in several developing countries as being ~10–12%, with women having a higher rate of disability than men. Recent efforts by the Washington City Group have yielded an activity-based screening instrument and other measures of disability that may well be the first step towards developing an activity-based global standard measurement of disability [10].

South Sudan is one of the most economically disadvantaged regions in the world and its health facilities are extremely scant [11]. In addition to ongoing economic hardship, South Sudan experienced a 21-year period of armed conflict. The signing of the Comprehensive Peace Agreement in 2005 ended the extensive war-related violence and large-scale forced displacement of individuals and resulted in the creation, in 2011, of the new nation of South Sudan. Despite this positive pattern of change, the growing influx of returnees to South Sudan has placed an extraordinary strain on already scant services and resources.

Given the lack of information on disability and factors that are associated with disability in post-conflict South Sudan, as well as the need for additional research on the long-term effects of exposure to traumatic events on disability, a survey was conducted in four states in the Greater Bahr el Ghazal region of South Sudan.

The aim of the study was to:

• Establish the prevalence rate of disability

• Estimate the prevalence rate of self-reported traumatic exposure of participants

• Investigate the gender differences in the association between exposure to traumatic events and disability, controlling for socio-demographic factors.

## Method

**Figure 1:**
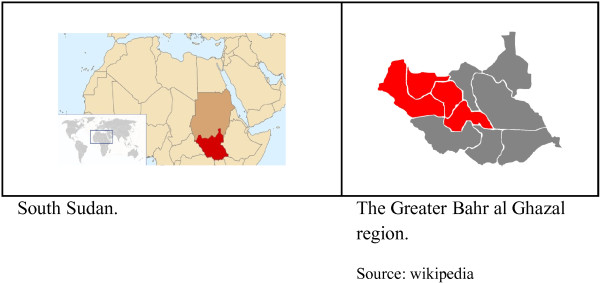
South Sudan and the Greater Bahr al Ghazal states.

A cross-sectional community survey was conducted in the Greater Bahr el Ghazal region of South Sudan in 2010. The Greater Bahr el Ghazal region consists of the following four states: Northern Bahr el Ghazal, Western Bahr el Ghazal, Lakes, and Warrap (Figure 1). It borders the Central African Republic to the west and Sudan to the north and has an estimated population of three million. The major part of the area is covered by swamps and ironstone plateaus. The region is populated by different ethnic groups: Dinka is the major one and other ethnic groups are Blanda, Jur/ Lou, Nuer, Bari and Zande [12]. The population in the region is predominantly rural with some variety within the four states; 92% of the population in Northern Bahr el Ghazal is classified as rural, compared to 57% in Western Bahr el Ghazal. Besides English which is the official language and Arabic which is spoken widely in the region, Dinka, Blanda, Jur/ Lou, Nuer, Bari and Zande are the spoken indigenous languages [12,13].

The sample frame was the general population of the four states in the Greater Bahr el Ghazal region. A multistage random cluster sampling method was used. The four states with 156 administrative units (‘Boma’) were divided in thirty survey clusters (our primary sample units). Highly politically insecure areas were not included in the survey. Nine bomas were randomly selected among the thirty clusters. The population data were based on the 2008 Sudan census [13]. These data were considered the most accurate population data available. The bomas were of different population size. The cluster selection was proportional to relative population size of each boma to ensure that each boma had the same probability of selection. We estimated the design effect at 2 to compensate for cluster randomization and the sample size was increased to double. In the next stage, the “spin-the-pen” method from the WHO Expanded Programme on Immunization [14] was used for household selection: the approximate geographic center of the area was identified and one household along an imaginary line connecting the center to the periphery was selected at random. Subsequent households were then selected by visiting every third closest household. Within each selected household, individuals who were 18 years or older and gave informed consent to take part in the study were assigned a number. A card was drawn at random from a deck of cards with corresponding numbers. The randomly selected household member was then interviewed. Individuals who were not able or declined to give informed consent or were visibly intoxicated were excluded from the study.

The participants were interviewed by health personnel (n= 11, five women and six men) from the region who were familiar with the cultural traditions and fluent in relevant local languages. They participated in two rounds of training workshops (9 days) prior to the data collection, during which the interviewers were trained in using the survey instruments. Furthermore, the cultural acceptability of the interview protocol was also discussed. The research instruments were available both in English and Arabic, but the main language used was Arabic which is widely used in the area. In addition, the key terms of the questionnaire were discussed and translated to indigenous languages of the area to ensure that the interviewers could easily explain all the items to the participants. Each household was approached by both a male and a female interviewer to ensure the interviewer’s gender would match that of the participant. In case of identifying any psychopathology with urgent treatment need amongst the participants, the interviewer referred the subject to an associated health provider. A total of 1236 households were contacted from which 1200 participants were recruited. The response rate was 95%.

Ethical clearance was obtained from the Research Department in the Ministry of Health of the Government of South Sudan and the Norwegian Regional Committee for Medical and Health Research Ethics. The local community leaders were informed about the aim and procedures of the study.

### Instruments

A questionnaire consisting of socio-demographic questions, including those pertaining to gender, age, marital status, education, employment situation, income regularity and household income, was administered to all participants. Because of the high influx of returnees to the region of study [15], the participants were also asked whether they were returnees. A returnee was defined as a person who had left his/her place of origin (regardless of the reason), but who had since returned to his/her place of origin.

Disability was measured using the Washington Group Short Measurement Set on Disability, which is an activity-based scale derived from the International Classification of Disability, Functioning and Health (ICF) [16]. This question set has been used for cross-cultural comparison of most commonly occurring disability domains after field trials and studies in several countries [17], including southern African countries [18]. It covers six functional domains or basic actions seeing, hearing, walking, cognition, self-care and communication—via the following questions.

1. Do you have difficulty seeing, even if wearing glasses?

2. Do you have difficulty hearing, even if using a hearing aid?

3. Do you have difficulty walking or climbing steps?

4. Do you have difficulty remembering or concentrating?

5. Do you have difficulty with self-care, such as washing all over or dressing?

6. Using your usual (customary) language, do you have difficulty communicating, for example understanding or being understood?

The response to each question was graded on a four-point scale: ‘no difficulty’, ‘some difficulty’, ‘a lot of difficulty’ or ‘unable to do’. The participants’ scores on these questions were calculated in three different ways, resulting in three forms of obtaining the prevalence of disability [18]:

1. Mild-to-severe disability: if ‘any difficulty’ in at least one of the six domains;

2. Moderate-to-severe disability: if ‘a lot of difficulty’ or ‘unable to do’ in at least one of the six domains;

3. Severe disability: if ‘unable to do’ in at least one of the six domains.

“Mild-to-severe disability” represents a broad definition of disability and “Severe disability” is the most limited [19]. Disability status as the dependent variable was dichotomised according to these three cut-offs. The Harvard Trauma Questionnaire (HTQ) was used to assess participants’ exposure to traumatic events. The HTQ is a widely used instrument that includes questions about traumatic events (40 events). The HTQ has been adapted for, and used in, various cultures and languages [20] including Sudan [21] and South Sudan [22]. The validity and reliability of the HTQ have been extensively tested in several settings and the instrument had good reliability and validity [20,23,24]. The Arabic version of the HTQ was employed in this study, after minor adaptations for the specific traumatic events of the South Sudan setting. The participants were asked to confirm or disconfirm exposure to each traumatic events (40 items) a) during the civil war (from 1983 to 2005) and b) after the Peace Agreement (after 2005). This gave us the opportunity to assess both recent and older traumatic experiences, which may differ in character. To facilitate the comparison of our findings with those of other relevant studies, we used 16 traumatic events from the 40-items list of HTQ in our analysis. These 16 traumatic events were identical to those applied by Roberts et al. [22] in their study of exposure to traumatic events among the population of Juba, South Sudan, and included: lack of food, water or shelter, combat situation (explosions, artillery fire, shelling and landmines), murder of family/friend, forced separation from family, rape or sexual abuse, forced isolation from others, being abducted or kidnapped, unnatural death of family/friend, very ill without medical care, being close to death, serious injury, tortured or beaten, murder of stranger or strangers, forced to accept thoughts against will, imprisonment. We also applied the cut-off points used by Roberts et al. [22] for the number of exposures to previous and recent traumatic events (cut-off points of eight and four events, respectively); hence, we treated exposure to traumatic events as a dichotomised variable.

Internal reliability was evaluated using Cronbach-alpha and estimated at 0.84 and 0.82 (for men and women respectively) for Washington Group Short Measurement Set on Disability. For HTQ (during the War) Cronbach-alpha was 0.75 and 0.76 (men and women respectively) and for HTQ (after the Peace Agreement) Cronbach-alpha was 0.86 and 0.84 (men and women respectively) which was above the commonly accepted level of 0.70 [25].

### Statistical analyses

Data analyses were conducted using SPSS (PASW) 18.0. Descriptive analyses were used to assess frequency of disability and exposure to traumatic events. We also used chi-square analysis to examine possible differences in exposure of traumatic events among disabled and non-disabled group. Logistic regression analyses were conducted to assess possible associations between various independent variables, including demographic factors and traumatic events, and the dependent variable (disability status). Subsequent analyses were performed stratified by gender in order to examine probable gender-specific differences. Separate hierarchical regression analyses were conducted to determine the relationship between independent variables and disability status, using a two-steps model with two blocks of independent variables. In the first step, socio-demographic variables that were significantly associated with disability in the bivariate analysis were entered into the model. These were age, marital status, employment status, household income, and being a returnee. Exposure to traumatic events was entered in the second and final step, which allowed the examination of the significance of exposure to traumatic events in predicting disability, while controlling for socio-demographic variables. The regression analysis was repeated for all three levels of disability: “Mild-to-severe disability”, “Moderate-to-severe disability”, and “Severe disability”.

## Results

### Socio-demographics and prevalence

Table 1 shows the demographic characteristics of the participants separated by gender and disability status (“Moderate-to-severe disability”). The participants were 56.4% male and 43.6% female. The corresponding rate for South Sudan is 51.8% male and 48.2% female [11]. Most participants were married (65.9%), with 18.3% of the total sample living in a polygamous marriage. In terms of education, 24.6% of men and 51.5% of women reported having no formal education. The overall estimated literacy rate for the country is 40% for males compared to 16% for females [11]. Only 28.8% of the participants had a regular household income. Thirty-three per cent of the participants reported being a returnee. The returnees-group had a higher proportion of men compared with the non-returnee participants (60% vs 53.8%; χ^2^ = 4.346, *P* < 0.05). The other socio-demographic characteristics of the returnees were mostly similar to those of the non-returnee participants. Participants living in a polygamous marriage constituted 18.3% of the sample and, compared with other participants in our sample, tended to be female (56.7% vs 40.3%; χ^2^ = 18.619, *P* < 0.01), older (mean age, 39.5 years; 95% CI [37.9–41.2] vs 33.6 years; 95% CI [32.8–34.33]; F = 6.244, *P* < 0.01) and have a lower level of education (62.4% of participants in a polygamous marriage never attended school vs 31.3% in the remainder of the sample; χ^2^ = 83.350, *P* < 0.01).

**Table 1:** Characteristics of participants by gender and disability status (“Moderate-to-severe disability”), N (%)

		**Total**	**Male**			**Female**		
			**Total**	**Not Disabled**	**Disable**	**Total**	**Not Disabled**	**Disable**
	Total	1200 (100.0)	660 (56.4)	562 (85.3)	97 (14.7)	510 (43.6)	451 (88.4)	59 (11.6)
Age (years)								
	18–25	308 (25.7)	164 (25.1)	140 (85.9)	23 (14.1)	136 (27.2)	128 (94.1)	8 (5.9)
	26–35	391 (32.6)	218 (33.4)	196 (89.9)	22 (10.1)	164 (32.8)	145 (88.4)	19 (11.6)
	36–50	395 (33.4)	216 (33.1)	184 (85.2)	32 (14.8)	167 (33.4)	143 (85.6)	24 (14.4)
	>50	89 (7.5)	55 (8.4)	36 (65.5)	19 (34.5)	33 (6.6)	25 (75.8)	8 (24.2)
Marital status								
	Single	320 (27.2)	237 (36.5)	203 (85.7)	34 (14.3)	81 (16.3)	75 (92.6)	6 (7.4)
	Married (one wife)	559 (47.6)	302 (46.5)	269 (89.4)	32 (10.6)	240 (48.3)	222 (92.5)	18 (7.5)
	No longer married	81 (6.9)	20 (3.1)	17 (85.0)	3 (15.0)	57 (11.5)	43 (75.4)	14 (24.6)
	Living in polygamous marriage	215 (18.3)	91 (14.0)	65 (71.4)	26 (28.6)	119 (23.9)	101 (84.9)	18 (15.1)
Education								
	Secondary school or higher	387 (32.8)	288 (44.6)	249 (86.5)	39 (13.5)	95 (18.8)	87 (91.6)	8 (8.4)
	Primary school	359 (30.4)	199 (30.8)	172 (86.9)	26 (13.1)	150 (29.7)	132 (88.0)	18 (12.0)
	Did not attend school	434 (36.8)	159 (24.6)	132 (83.0)	27 (17.0)	260 (51.5)	227 (87.3)	33 (12.7)
Employment								
	Paid work	291 (26.5)	199 (32.9)	177 (89.4)	21 (10.6)	88 (19.0)	76 (86.4)	12 (13.6)
	Self-employed	422 (38.5)	234 (38.7)	205 (87.6)	29 (12.4)	169 (36.4)	153 (90.5)	16 (9.5)
	Student	1448 (13.1)	101 (16.7)	84 (83.2)	17 (16.8)	42 (9.1)	39 (92.9)	3 (7.1)
	Non paid work	129 (11.8)	32 (5.3)	24 (75.0)	8 (25.0)	96 (20.7)	86 (89.6)	10 (10.4)
	Unemployed	111 (10.1)	38 (6.3)	28 (73.7)	10 (26.3)	69 (14.9)	56 (81.2)	13 (18.8)
Regular income								
	No	823 (70.4)	396 (62.2)	340 (85.9)	56 (14.1)	402 (79.9)	85 (84.2)	16 (15.8)
	Yes	346 (29.6)	241 (37.8)	202 (84.2)	38 (15.8)	101 (20.1)	445 (88.5)	58 (11.6)
Household monthly income (US dollars)								
	<75	553 (63.1)	286 (54.6)	233 (81.5)	53 (18.5)	247 (75.3)	213 (86.2)	34 (13.8)
	75–200	209 (23.9)	158 (30.2)	142 (89.9)	16 (10.1)	49 (14.9)	41 (83.7)	8 (16.3)
	200–350	85 (9.7)	60 (11.5)	50 (84.7)	9 (15.3)	23 (7.0)	18 (78.3)	5 (21.7)
	>350	29 (3.3)	20 (3.8)	17 (85.0)	3 (15.0)	9 (2.7)	9 (100.0)	0 (0.0)
Returnee								
	No	781 (66.9)	414 (65.1)	358 (86.7)	55 (13.3)	356 (70.9)	326 (91.6)	30 (8.4)
	Yes	386 (33.1)	222 (33.6)	187 (84.2)	35 (15.8)	146 (29.1)	118 (80.8)	28 (19.2)
Trauma exposure during the war >8 #								
	< 8	893 (74.4)	461 (69.8)	409 (88.9)	51 (11.1)	405 (79.4)	374 (92.3)	31 (7.7)
	>=8	307 (25.6)	199 (30.2)	153 (76.9)	46 (23.1)	105 (20.6)	77 (73.3)	28 (26.7)
Trauma exposure after the Peace Agreement > 4 ¤								
	< 4	1079 (90.2)	582 (88.6)	511 (88.0)	70 (12.0)	467 (91.7)	420 (89.9)	47 (10.1)
	>=4	117 (9.8)	75 (11.4)	48 (64.0)	27 (36.0)	42 (8.3)	30 (71.4)	12 (28.6)

# Range of traumatic events: 0–16.

¤ Range of traumatic events: 0–8.

Table 2 shows the prevalence of disability among the study sample according to three different estimates. Using a conservative disability threshold (“Severe disability”), only 3.6% of the population was identified as having a disability. In contrast, the prevalence of disability was 40.5% for “Mild-to-severe disability” , whereas the use of the intermediate estimate (“Moderate-to-severe disability”) as the cut-off for identifying disability revealed that 13.4% of participants had reported a disability. The overall prevalence of disability tended to increase with age across all three disability thresholds. The only exception in this pattern was men in the age group 18–25 years who showed a higher rate of disability than men in the age group 26–50 years: for instance regarding “Moderate-to-severe disability” 14.1% of men in the age group 18–25 were disabled while 10% and 14.8% were disabled in age groups 26–35 and 36–50 respectively (χ^2^ = 20.986, *P* < 0.01). The differences in disability prevalence between men and women were not statistically significant. A significantly higher percentage of disabled individuals (with “Moderate-to-severe disability”) lived in a polygamous marriage compared with non-disabled participants: 28.6% and 12.4% respectively (χ^2^ = 15.445, *P* < 0.01) for men and 15.1% and 10.1% respectively (χ^2^ = 5.464, *P* < 0.01).

**Table 2:** Estimation of the prevalence of disability based on three thresholds, stratified by gender

	**Total**	**Male N (%)**	**Female N (%)**	**χ2**
Mild-to-severe disability: ‘some difficulty’ in at least one of the six domains	486 (40.5)	261 (39.6)	211 (41.4)	0.373 ns
“Moderate-to-severe disability” : ‘a lot of difficulty’ or ‘unable to do’ in at least one of the six domains	161 (13.4)	97 (14.7)	59 (11.6)	2.468 ns
“Severe disability”: ‘unable to do’ in at least one of the six domains	43 (3.6)	27 (4.1)	16 (3.1)	0.748 ns

*p < 0.05.

§ Missing value: N=1, 01%.

Almost all participants (95.3%) reported having been exposed to at least one type of traumatic event during the war (mean, 5.9 events; 95% CI [5.60–6.10]). For the period after the Peace Agreement, 26.6% of individuals reported exposure to at least one type of traumatic event (mean, 0.96 events; 95% CI [0.83–1.10]). Men reported higher rate of traumatic event exposure compared to women.

Disabled participants reported higher rate of exposure to trauma events, both during the war and after the Peace Agreement, compared to non-disabled. This pattern was observed for all three disability thresholds. We also observed differences between the disabled and non-disabled in the type of traumatic events they reported. For instance, about 44% of disabled participants reported exposure to combat situation (explosions, artillery fire, shelling and landmines) compared with about 20% among non-disabled participants (χ^2^ = 46.69 for “Moderate-to-severe disability”, *P* < 0.01). The differences between disabled and non-disabled participants regarding the rate of exposure to traumatic events were significant for all event types with the exception of the following (for which disabled and non-disabled individuals did not exhibit a significant difference regarding exposure rate): forced separation, being close to death, and kidnapped (for traumatic events during the war). Regarding the traumatic events after the Peace Agreement, disabled participants reported significantly higher rate of exposure for all types of traumatic events with the following exceptions: the rate of experiencing sexual abuse or rape was not statistically different between disabled and non-disabled participants (“Severe disability”). Disabled participants (“Mild-to-severe disability”) did not differ with non-disabled participants in reporting of the following experiences: lack of food, water and shelter, exposure to combat situation, being close to death.

The most frequently reported traumatic events were similar between disabled and non-disabled participants (“Moderate-to-severe disability”), as follows: during the war: lack of food and water (80.7% and 71.8%, respectively); lack of shelter (73% and 66.3%, respectively); and suffered ill health without access to medical care or medicine (71.4% and 58.4%, respectively). Regarding exposure to traumatic events in recent times (after the Peace Agreement): lack of food and water (18% and 9.6%, respectively); lack of shelter (17.4% and 6.2%, respectively); and forced isolation from others (18% and 10.6%, respectively).

A hierarchical logistic regression analysis was used to calculate adjusted odds ratios and to cast light on the relationship between disability (“Moderate-to-severe disability”), socio-demographic factors, and exposure to traumatic events. The analysis was performed separately for men and women. Table 3 shows the odds ratios for the various variables in the model. The final step in the regression showed that, for male participants, exposure to traumatic events both during the war and after the Peace Agreement was a risk factor for having a disability when the socio-demographic factors were taken into account. For women, experiencing a high level of traumatic events (more than eight traumatic events) during the war was significantly associated with being disabled. The trauma experiences after the Peace Agreement did not remain statistically significant as a risk factor for being disabled among women. In addition, Being older than 50 years and living in a polygamous marriage increased the likelihood of having a disability for both men women.

**Table 3:** Results of hierarchical regression analysis with demographic factors and exposure to trauma as independent variables and Disability (Moderate-to-severe disability) as the dependent variable

		**Step 1:Socio-demographic variables. Adjusted odds ratio [95% CI]**		**Step 2: Socio-demographic variables and exposure to traumatic events. Adjusted odds ratio [95% CI]**	
		**Male**	**Female**	**Male**	**Female**
Age (years)					
	18–25	1	1	1	1
	26–35	0.690 (0.290–1.645)	1.791 (0.619–2.181)	0.693 (0.276–1.738)	1.953 (0.645–2.910)
	36–50	1.490 (0.562–3.949)	1.138 (0.370–3.497)	1.876 (0.670–5.253)	1.274 (0.395–4.109)
	>50	4.067 (1.228–6.469)*	5.824 (2.228–7.068)*	6.217 (1.709–8.615)*	7.930 (3.441–8.657)*
Marital status					
	Single	1	1	1	1
	Married (one wife)	0.698 (0.296–1.643)	1.735 (0.426–3.070)	0.620 (0.256–1.503)	1.480 (0357–4.141)
	No longer married	0.601 (0.067–5.398)	4.651 (2.311–5.735)*	0.855 (0.094–5.808)	5.194 (0.973–6.725)
	Living in polygamous marriage	3.031 (1.129–5.035)*	3.321 (2.460–4.360)*	2.502 (1.890–6.032)*	5.029 (3.132–5.347)*
Employment					
	Paid work	1	1	1	1
	Self-employed	1.204 (0.537–2.699)	1.192 (0.398–3.572)	1.426 (0.623–3.262)	1.275 (0.403–3.034)
	Student	3.842 (1.281–5.519)*	1.329 (0.199–3.861)	3.558 (1.138–4.120)*	0.533 (0.134–2.298)
	Non paid work	3.315 (1.028–4.693)*	0.465 (0.119–1.821)	3.017 (0.878–4.370)	1.189 (0.301–2.701)
	Unemployed	3.863 (1.148–8.998)*	1.405 (0.382–5.160)	3.320 (0.901–9.234)	1.378 (0.614–3.093)
Household income					
	>350	1	1	1	1
	200–350	0.684 (0.318-1.473)	1.261(0.395-4.019)	0.692 (0.318–1.506)	1.120 (0.342–1.661)
	75–200	1.240 (0.460–3.338)	2.945 (0.575–4.080)	1.217 (0.436–3.398)	3.315 (0.620–4.717)
	<75	1.409 (0.333–5.967)	0.000	1.486 (0.309–6.151)	0.000
Returnee					
	No	1	1	1	1
	Yes	0.925 (0.513–1.669)	0.925 (0.513–1.669)	0.657 (0.345–1.252)	1.378 (0.614–3.093)
Trauma exposure during the war>8 #					
	No			1	1
	Yes			3.243 (1.228–4.097)*	3.998 (1.744–5.161)*
Trauma exposure after the Peace Agreement > 4					
	No			1	1
	Yes			4.800 (1.769–8.164)*	1.472 (0.538–3.026)

*p < 0.05.

# Range of traumatic events: 0–16.

¤ Range of traumatic events: 0–8.

Identical regression analyses were conducted using the other two disability thresholds (“Mild-to-severe disability” and “Severe disability”). Table 4 shows the result of the three separate regression analyses, with “Mild-to-severe disability”, “Moderate-to-severe disability”, and “Severe disability” as three different independent variables. Regarding “Severe disability”, recent traumatic events (more than 4 trauma experience after the Peace Agreement) emerged as a risk factor for disability in both men and women. For women, being self-employed was also significantly associated with being disabled. Defining disability with a low threshold (“Mild-to-severe disability”), we observed different sets of risk factors for men and women: for women, being older than 36 years increased the odds of being disabled. While among men, the age factor was limited to older than 50 years. Being a returnee increased the likelihood of having disability only among women. Experiencing recent traumatic events was a risk factor only for men. In addition, having an unpaid job was significantly associated with having disability among men.

**Table 4:** Summary of statistically significant results of multiple regression analysis (adjusted), for male and female, with demographic factors and exposure to trauma as independent variables and Disability (Mild-to-severe disability, Moderate-to-severe disability, and Severe disability) as the dependent variables

	**Mild-to-severe disability**		**Moderate-to-severe disability**		**Severe disability**	
	**Male**	**Female**	**Male**	**Female**	**Male**	**Female**
Age: 36–50 years		2.174 (1.053–2.194)				
Age: >50 years	3.040 (1.169–5.904)	6.777 (1.52–8.369)	6.217 (1.709–8.615)	7.930 (3.441–8.657)		
Living in polygamous marriage			2.502 (1.890–6.032)	5.02 (3.132–7.347)		
Self-employed						2.341 (1.083–3.816)
Student			3.558 (1.138–4.120)			
Non paid work	6.979 (2.593–8.783)					
Returnee		1.044 (0.588–1.854)				
Trauma exposure during the war >8 #	1.589 (1.034–2.444)		3.243 (1.228–4.097)	3.998 (1.744–5.161)		
Trauma exposure after the Peace Agreement > 4 ¤			4.800 (1.769–8.164)		7.269 (2.382–9.177)	7.049 (2.093–9.445)

Only statistically significant (*p < 0.05) associations are presented.

# Range of traumatic events: 0–16.

¤ Range of traumatic events: 0–8.

Possible interactions between independent variables were examined and no significant associations between independent variables were found.

## Discussion

A disability prevalence rate of 13.4% was obtained in this study (“Moderate-to-severe disability”). A study performed in Zambia applying the same disability measurement and cut-off used in our study reported a disability rate of 11% (for age group 15–65) [18]. One would have expected a greater difference between the rate of disability obtained in our study and that of the Zambian study, with a higher rate of disability in the war-affected setting of South Sudan. However, as traumatic-event exposure was not examined in the Zambian study, a direct comparison is not possible. One explanation for the similarity in disability rates observed may be the possible over-reporting of disability in the Zambian study and/or the possible under-reporting of disability in our study. Arguably, after being exposed to the hardship of long-term armed conflict, our participants were less likely to report disability. Such potential under-reporting and resilience factors should be investigated in future research.

No significant differences were observed in disability prevalence between men and women. Women have been reported to have slightly higher disability prevalence in other low and middle income countries [9]. This difference is explained as being partly due to the fact that women live longer, and disability is strongly correlated with age. In Loeb and Eide’s study in Zambia, however, the rate of disability was higher among men [26].

Women living in a polygamous marriage had increased odds of being disabled (with “Moderate-to-severe disability”) after controlling for other variables, including exposure to traumatic events. The literature regarding how marital status influences the association between exposure to traumatic events and disability is scarce. However, some studies performed in sub-Saharan Africa showed that women in a polygamous marriage experience a higher degree of mental distress compared with women in a monogamous marriage [27,28] and report reduced life satisfaction, higher rates of domestic physical and sexual abuse and greater prevalence of low self-esteem [28]. Other studies found no association between the symptoms of either anxiety or depression and polygamy (e.g. [29]). Disabled women may tend to be in polygamous marriages because of their lower social status. Living in a polygamous marriage may also have a disabling effect on the individual. Similarly, our results indicated that men with more than one wife showed increased odds of being disabled after controlling for other variables compared with men in a monogamous marriage. Little is known about how polygamous marriage, compared with monogamous marriage, affects the life satisfaction and mental distress of men.

Returnees might be considered as a risk group for disability [30]; however, we did not find that being a returnee was significantly associated with having a disability after controlling for the other variables. One explanation is that the returnees’ characteristics, in our sample, were similar to the not-returnee group.

The range of traumatic-event exposure in our study was in accordance with those reported in studies performed in other post-conflict settings [31-33] and in Juba, South Sudan [22]; the elevated level of reported traumatic events confirms the results of the study of Roberts et al. [22]. The decrease in reported traumatic events for the period after the Peace Agreement was encouraging and expected. This study showed that experiencing traumatic events was significantly associated with disability: for instance, the odds of being disabled (“Severe disability”) for men and women who had experienced high number of recent traumatic events were about 7 times that of participants with fewer traumatic exposures. In addition, traumatic experiences during the War increased the likelihood of having a disability among men and women (Moderate-to-severe disability). Compared with non-disabled participants, participants with disability were more likely to have experienced higher number of traumatic events during the war and after the Peace Agreement. The association between exposure to traumatic events and disability has been documented in conflict-affected settings [1,34-36], among resettled refugee populations [34,37], and in post-conflict settings [38]. Therefore, the results of our study are consistent with previous findings. For instance, the study of Momartin et al. [34], which was performed among Bosnian refugees in Australia, showed that some traumatic events predicted both severity of posttraumatic stress disorder (PTSD) and impairment in psychosocial functioning, whereas other traumatic events were not associated with PTSD status or level of functional impairment. Miller et al. [39] studied the association between traumatic-event exposure and functional impairment in a post-war setting. The impact on health outcomes (psychological distress, PTSD and functional impairment) of previous war trauma was compared with that of daily stressors. The results of that analysis showed that daily stressors had a direct effect on functional impairment, whereas war experiences did not. In a population-based mental health survey, Cardozo et al. [33] investigated mental health status, level of functioning, and traumatic-event experience among disabled and non-disabled Afghans in post-war Afghanistan. Increased exposure to traumatic events was associated with poorer social functioning among both disabled and non-disabled participants.

The finding that exposure to combat situation (explosions, artillery fire, shelling and landmines) was reported more frequently among the disabled participants (“Moderate-to-severe disability” and “Severe disability”) may indicate that they had become disabled as a consequence of the war. However, the results of our study may also be interpreted differently: individuals with a pre-existing disability may have a greater risk of experiencing traumatic events.

The disabled participants in our study reported a significantly higher rate of exposure to most of the traumatic events compared with non-disabled individuals. However, the traumatic events reported most frequently were similar in both groups. These findings are consistent with the results reported by Cardozo et al. [33], which showed that disabled individuals reported a higher rate of exposure to traumatic events than did non-disabled participants. Similarly to our findings, lack of food, water and shelter and ill health without access to medical care or medicine were among the traumatic experiences reported most commonly in post-conflict Afghanistan.

This study demonstrates that it was possible to conduct such a community survey under very difficult circumstances. For example, there was a lack of proper infrastructure, making it difficult to reach some of the sampling areas, and the security situation had to be carefully and continuously monitored. Emergency psychiatric treatment was therefore occasionally provided by the article’s fourth author (a physician). A high response rate was obtained which is partly due to the community leaders’ approval of the study. The socio-demographic characteristics of our sample were comparable to those of the general population and similar to socio-demographic characteristics found in the Roberts et al. study in Juba [22]. This study had some limitations. As a cross-sectional study, it could not identify cause-and-effect relationships between the demographic factors studied and traumatic events and disability. The 2008 Sudan census, which was used as the source of population data and in the sampling process, has its inaccuracies particularly because of the large-scale migration process and the influx of returnees. In addition, the a priori exclusion of the insecure areas creates a bias, which is difficult to measure. Furthermore, we used self-reported measures to assess exposure to traumatic events, which may introduce a bias based on inconsistencies regarding the recall of events [40]. Self-reported measures rely on the participant’s memory and are prone to be influenced by dominating attitudes towards the themes of study. The use of an additive scale of traumatic events is a simple way of including an indicator of exposure. However, this would not differentiate between the types and the severity of the events. Finally, although the instruments used in this study have been used widely and internationally in various cultural settings, and the interviewers were familiar with the socio-cultural setting, no formal socio-cultural validation was conducted. The interviewers translated some of the words in the questionnaire into the indigenous languages. This was the case in about 20% of the interviews. The use of the indigenous languages was, however, not systematic measured and hence represents a source of bias. We were not able to formally assess inter-rater reliability. However, attempt was made, through repeated and supervised interview practice, to ensure a satisfactory level of rating agreement among the interviewers.

These limitations may influence the generalizability of the study results. We believe, however, the results are relevant for Greater Bahr el Ghazal States as well as for other post-conflict settings. The findings cast light on various aspects of the war-affected society of South Sudan, indicating the similarities and particularities of this setting compared with that of other post-conflict societies regarding the impact of war on the health of the population.

## Conclusions

Documenting the association between traumatic experience and disability has possible implications for the construction of disability services and provision of health services to individuals exposed to traumatic events. The risk factors for disability may help guide future disability and health planning in South Sudan, and should be considered in other low-income countries. The higher rates of trauma exposure among the disabled section of the population underline the risks to which individuals with disability are exposed during armed conflicts and their aftermath. The human rights implications of these and similar findings should be addressed by researchers, national authorities and NGOs, and investigated further [41].

## Competing interests

The authors declare that they have no competing interests.

## Authors’ contributions

TA: executed the statistical analysis and drafted the manuscript; participated in the design of study and drafting of the manuscript. LL: participated in the design of study and drafting of the manuscript. AHE: participated in the design of study and drafting of the manuscript. RJ: participated in the drafting of the manuscript. RAA: participated in the design of the study and data collection. EH: supervised, participated in the design of study and drafting of the manuscript. All authors read and approved the final manuscript.

## Pre-publication history

The pre-publication history for this paper can be accessed here:

http://www.biomedcentral.com/1471-2458/13/469/prepub
